# Abdominal Tuberculosis: A Diagnostic Dilemma

**DOI:** 10.5005/jp-journals-10018-1133

**Published:** 2015-01-06

**Authors:** Arun Joyati Tarafder, Mamun-Al Mahtab, Sisir Ranjan Das, Rezaul Karim, Habibur Rahaman, Salimur Rahman

**Affiliations:** 1 Department of Hepatology, Mymensingh Medical College, Mymensingh, Bangladesh; 2 Department of Hepatology, Bangabandhu Sheikh Mujib Medical University, Shahbag, Dhaka, Bangladesh; 3 Department of Neonatology, Dhaka Medical College, Dhaka, Bangladesh; 4 Upazila Health Complex, Dhamrai, Dhaka, Bangladesh

**Keywords:** Tuberculosis, Abdominal tuberculosis, Diagnosis.

## Abstract

According to the World Health Organization, more than 2 billion people are estimated to be infected with tuberculosis (TB) and approximately 95% of tuberculosis cases occur in developing countries. Extrapulmonary forms of tuberculosis constitute approximately one-sixth of all cases and the prevalence of extrapulmonary tuberculosis (TB) seems to be rising, particularly due to increasing prevalence of acquired immunodeficiency syndrome (AIDS). In patients with extrapulmonary TB, abdomen is involved in 12% of patients. Gastrointestinal involvement is found in 66 to 75% of abdominal cases, with the terminal ileum and the ileocecal region being the most common sites of involvement. Here, we report a case of abdominal TB.

**How to cite this article:**

Tarafder AJ, Al-Mahtab M, Das SR, Karim R, Rahaman H, Rahman S. Abdominal Tuberculosis: A Diagnostic Dilemma. Euroasian J Hepato-Gastroenterol 2015;5(1):57-59.

## INTRODUCTION

The patients, 25 years old man, normotensive, non-diabetic, from middle class socioeconomic background presented with abdominal pain, abdominal distention and fever for 1 month. He had no history of joint pain, hematemesis and/or melena, cough or breathlessness, chest pain or palpitation, facial puffiness and urinary complains. He never experienced jaundice and his bowel habit was normal. On clinical examination, the patient was ill looking, anemic, icteric, well oriented and cooperative with average built and nutrition. He had no bony tenderness, lymphadenopathy and any organomegaly. He had ascites and sluggish of bowel sound but liver dullness was not obliterated. He had no edema, flapping tremor and stigmata of chronic liver disease. Others systemic examination revealed no abnormality. Finally, the patient was admitted to a tertiary care hospital for proper evaluation.

Hematological investigations revealed hemoglobin 8.6 gm/dl, ESR-40 mm/lst hour, platelets 890 × 10^9^/l, and total count of WBC 41,100/mm^3^. Peripheral blood film showed microcytic hypochromic anemia and neutrophilic leukocytosis with thrombocytosis. He tested negative for HBsAg, anti-HCV, anti-HEV Ig M, and anti-HAV Ig M. His bilirubin level was 5.8 mg/dl, serum ALT 24 U/L, serum AST 44 U/L, serum alkaline phosphatase 75 IU/L, LDH 259 U/L and serum albumin 3.3 gm/l. He had normal prothrombin time and D-dimer level. His random blood sugar was 5.6 mmol/l, serum creatinine 0.8 mg/dl and uric acid level 6 mg/dl. He also tested negative for ANA, ASMA, RK39 and malarial parasite. He had CEA 1.97 ng/ml.

Ultrasonography and computed tomography (CT) scan of whole abdomen revealed hepatosplenomegaly and moderate ascites. Endoscopy, colonoscopy and chest X-ray revealed normal findings. A tuberculin skin test was negative and sputum were negative for AFB in three occasions. Ascitic fluid was taped and studied. Ascitic fluid showed lymphocytes 95%, negative for AFB, gram stain and malignant cell. His ascitic fluid protein 38 gm/l, sugar 6 mmol/l and ADA 22.6 U/l. Bone marrow examination was also done. It demonstrated panmyelosis. He had normal Hb electrophoresis study.

In spite of all investigations, definitive diagnosis could not be made. He was identified as a tuberculosis (TB) suspect and was started on treatment with anti-TB drug (CAT-1) and patient gradually felt better. He was subsequently discharge home to directly observed therapy (DOT). His fever improved over the initial weeks of therapy, ascites resolved and engaged himself with his normal daily activities. Two months later, repeat scans and laboratory tests are to be in normal range.

## DISCUSSION

Abdominal tuberculosis is known to human race since the times of Hippocrates.^1-7^ It is a common killer disease in underdeveloped countries and is being seen with increasing frequency in the western world.^1,2,8^ Abdominal TB is defined as *M. tuberculosis* infections in the gastrointestinal tract, peritoneum, or intra-abdominal solid organs.^5,6,9^ There are four different possible pathways for intra-abdominal *M. tuberculosis* infection: hematogenous spread from primary pulmonary TB, ingestion of infected milk products, ingestion of infected sputum from pulmonary TB and the direct invasion from an adjacent organ.^3,10^

Abdominal TB usually presents with vague, nonspecific symptoms, making diagnosis very difficult for clinicians. Physical exam is usually notable for abdominal tenderness, mass, ‘doughy abdomen,’ or hepatosplenomegaly. Laboratory values commonly exhibit anemia and an elevated ESR which are nonspecific.^11^ PPD skin test is positive in the majority of patients with abdominal tuberculosis, but it lacks diagnostic significance because of the high rate of false-positives.^12^

Radiological studies act as an adjunct in the diagnosis of abdominal tuberculosis. Computed tomography has proven to be an effective tool in recognizing the pathology consistent with abdominal tuberculosis although the findings can be nonspecific and may mimic many other conditions, including inflammatory bowel disease or colon cancer.^13^ The most common findings on CT that are highly suggestive of abdominal TB are high density ascites, lymphadenopathy, bowel wall thickening, and irregular soft-tissue densities in the omental area.^4,14^ Evidence of tuberculosis reported on chest X-ray in 46% of patients; chest X-ray is positive in 80% of patients when acute complications, such as obstruction, perforation and peritonitis are present.^15^

Ultrasonography being a widely available investigation is now a ‘low-threshold’ diagnostic procedure for all patients suspected to have abdominal TB. It can accurately demonstrate small quantities of ascitic fluid and is an effective method for detection of peritoneal disease. The reported findings include multiple, thin, complete and incomplete septae, visible echogenic debris seen as fine strands or particulate matter within the fluid.^16^ These strands of septae may be due to high fibrin content of the exudative ascitic fluid. Septae have also been reported in a few cases of malignant ascites.^17^

Endoscopy may be useful in cases of GI tuberculosis where lesions are accessible. Endoscopic appearances in tuberculosis include hyperemic nodular friable mucosa, irregular ulcers with sharply defined margins and undermined edges and pseudopolyps. These may mimic inflammatory bowel disease and malignancy. Endoscopic biopsy may not reveal granulomas in all cases, as the lesions are submucosal.^18^ Biopsies from the edges and the base of the ulcer or multiple biopsies from the same site may increase the yield. Endoscopic biopsy specimens may be subjected to polymerase chain reaction for detection of AFB.^19^ Colonoscopy has proven to be invaluable in the diagnosis of intestinal tuberculosis, due to its ability to identify the pathology both macroscopically and microscopically, via biopsy. Morphology of tubercular lesions has also been described as ulcerative, hyper-trophic and ulcerohypertrophic.^20^ Differentiation from Crohn’s disease can be difficult. Evidence of transverse ulcers, absence of crypt distortion, or extensive chronic inflammation in the lamina propria distant from ulcerated foci all suggest abdominal TB.^21^

Analysis of ascetic fluid often shows lymphocytic predominance with a serum-to-ascites albumin gradient of <1.1 gm/dl.^22^ The reported sensitivity of adenosine deaminase activity of tuberculous ascitic fluid varies.^23^ In noncirrhotic patients, adenosine deaminase activity (ADA) of >33 U/l in ascitic fluid is shown to have a sensitivity of 97% and specificity of 100% in TB peritonitis.^24^ The smear and culture of ascitic fluid have low diagnostic yield. A peritoneal biopsy should be done via laparoscopy or laparotomy to minimize any possible diagnostic delay. Thickened peritoneum, studding of the peritoneum with multiple tubercles and adhesions are often seen on laparo-scopy or laparotomy. Biopsy of these tubercles shows granulomatous changes. PCR testing of the biopsy tissue and culture allows rapid diagnosis of tuberculous peritonitis. Microbiological confirmation and/or histological appearance of granulomas, with or without caseation, establishes the diagnosis.^25^
